# Supplementary figures and images for: Gut microbiome mediates the associations between lifestyle factors and risk of colorectal high-risk adenoma: results from a population-based cohort study
Source: mSystems. 2025 Sep 22;10(10):e00933-25. doi: 10.1128/msystems.00933-25 (PMC12542775; doi:10.1128/msystems.00933-25)

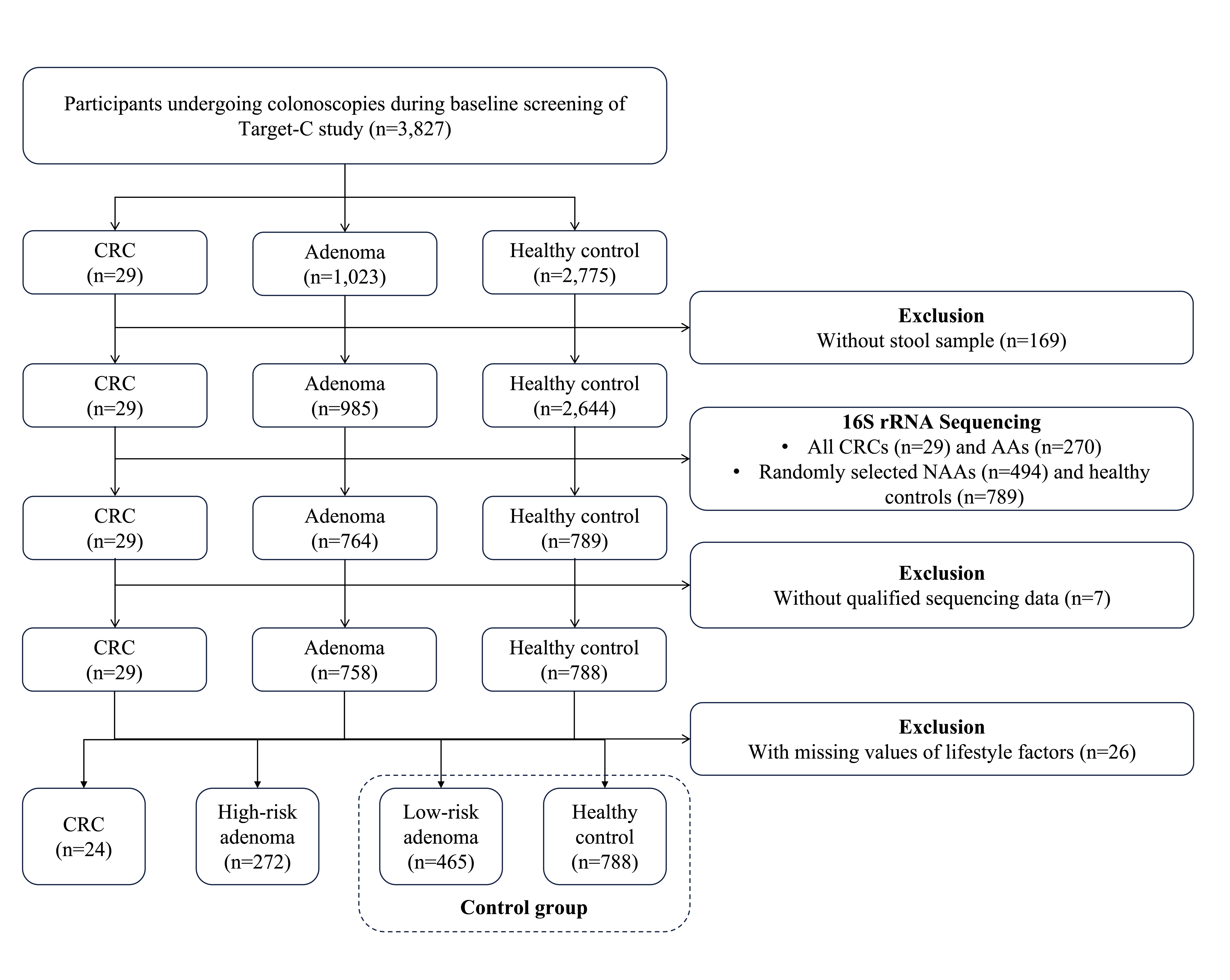

Supplement: Figure S1 — Workflow diagram for subject selection. [file msystems.00933-25-s0001.tiff]
