# Supplementary material for: Gut microbiome mediates the associations between lifestyle factors and risk of colorectal high-risk adenoma: results from a population-based cohort study
Source: mSystems. 2025 Sep 22;10(10):e00933-25. doi: 10.1128/msystems.00933-25 (PMC12542775; doi:10.1128/msystems.00933-25)
Supplement: Supplemental Material — Supplemental methods, figure legend, and tables. [file msystems.00933-25-s0002.docx]

# Supplementary Material

**Methods**

**External cohort**

## To validate the correlation between genera and lifestyle factors, we further collected published fecal whole metagenome sequencing data from Japanese cohorts(1), consisting of patients with colorectal cancer and healthy controls, covering a range of samples. Raw sequencing data of these samples were downloaded from Sequence Read Archive (SRA) using the following accession IDs: DRA006684 and DRA008156. The metadata were manually curated from relevant original publication. Taxonomic profiles of shotgun metagenomes were generated using MetaPhlan4, which uses a library of clade-specific markers to provide pan-microbial profiling(2). Only colorectal cancer samples and healthy controls were included for downstream integrative analysis.

## We first retrieved data from CRC patients and healthy controls with complete information on alcohol consumption, body mass index, and smoking (Brinkman index) using the ‘curatedMetagenomicData’ R package, then performed 1:1 nearest matching by age, gender, and body mass index. As a result, we obtained data from 299 CRC patients and 299 healthy controls, including information on age, gender, BMI, disease diagnosis, smoking, and alcohol consumption. We then matched their fecal metagenome sequencing data, creating our external validation cohort.

## In this external validation, we used the same approach as in our own cohort analysis, employing the multivariate association with linear models (MaAsLin2) method to analyze the association between gut microbiota and lifestyle factors. However, due to the lack of data types, we only adjusted for age, gender, BMI, and disease status in the external validation.

## Table and Figure Legends

## TABLE S1 Multivariate association between gut microbiota and lifestyle factors using MaAsLin2 Analysis.

## TABLE S2 Characteristics of the study population in external cohort.

## TABLE S3 Multivariate association between gut microbiota and lifestyle factors using MaAsLin2 Analysis in external cohort.

## Fig S1 Workflow diagram for the subject selection.

## AA: advanced adenoma; CRC: colorectal cancer; NAA: non-advanced adenoma.

## TABLE S1 Multivariate association between gut microbiota and lifestyle factors using MaAsLin2 Analysis.

| **Genus^#^** | **Lifestyle factor** | **Coefficient** | **P value^*^** | **FDR** |
| --- | --- | --- | --- | --- |
| **Phylum: *Actinobacteria*** | | | | |
| *g__Actinomyces* | Cigarette consumption | 0.266 | <0.001 | <0.001 |
| *g__Bifidobacterium* | Alcohol consumption | -0.297 | 0.002 | 0.080 |
| *g__Collinsella* | BMI | 0.236 | 0.011 | 0.158 |
| *g__Slackia* | Alcohol consumption | 0.137 | 0.007 | 0.137 |
| **Phylum: *Bacteroidetes*** | | | | |
| *g__Bacteroides* | BMI | -0.186 | 0.005 | 0.130 |
| *g__Odoribacter* | BMI | -0.137 | 0.014 | 0.180 |
| *g__Butyricimonas* | BMI | -0.178 | <0.001 | 0.020 |
| *g__Prevotella* | BMI | -0.071 | 0.008 | 0.137 |
| *g__Prevotella 9* | Alcohol consumption | 0.533 | 0.001 | 0.060 |
| *g__Alistipes* | BMI | -0.265 | <0.001 | 0.017 |
| *g__Parabacteroides* | BMI | -0.188 | 0.005 | 0.130 |
| **Phylum: *Firmicutes*** | | | | |
| *g__Weissella* | BMI | 0.143 | 0.022 | 0.234 |
| *g__Streptococcus* | Alcohol consumption | -0.195 | 0.015 | 0.191 |
| *g__Clostridium sensu stricto 1* | Alcohol consumption | 0.235 | 0.011 | 0.158 |
| *g__Eubacterium ventriosum group* | Alcohol consumption | -0.188 | 0.013 | 0.178 |
| *g__Eubacterium xylanophilum group* | Alcohol consumption | -0.134 | 0.009 | 0.146 |
| *g__Ruminococcus gnavus group* | Cigarette consumption | 0.304 | 0.012 | 0.166 |
| *g__Agathobacter* | BMI | -0.101 | 0.004 | 0.125 |
| *g__Hungatella* | BMI | -0.181 | <0.001 | 0.013 |
| *g__Lachnospiraceae FCS020 group* | Alcohol consumption | -0.164 | 0.009 | 0.141 |
| *g__Lachnospiraceae NK4A136 group* | BMI | -0.183 | 0.023 | 0.236 |
| *g__Lachnospiraceae UCG 003* | Cigarette consumption | -0.126 | 0.012 | 0.164 |
| *g__Tyzzerella 3* | Alcohol consumption | -0.255 | 0.003 | 0.095 |
| *g__Tyzzerella 3* | BMI | 0.187 | 0.014 | 0.182 |
| *g__Tyzzerella 4* | Alcohol consumption | 0.224 | 0.020 | 0.224 |
| *g__Flavonifractor* | BMI | -0.130 | 0.023 | 0.236 |
| *g__Negativibacillus* | Alcohol consumption | 0.204 | <0.001 | 0.017 |
| *g__Oscillibacter* | BMI | -0.211 | <0.001 | 0.020 |
| *g__Ruminiclostridium 5* | Alcohol consumption | -0.143 | 0.009 | 0.137 |
| *g__Ruminococcaceae UCG 010* | Alcohol consumption | -0.112 | 0.002 | 0.090 |
| *f__Ruminococcaceae; g__UBA1819* | BMI | -0.172 | 0.002 | 0.087 |
| *g__Subdoligranulum* | Alcohol consumption | -0.297 | 0.010 | 0.153 |
| *g__Erysipelatoclostridium* | BMI | -0.145 | 0.005 | 0.133 |
| *g__Holdemanella* | Alcohol consumption | 0.167 | 0.018 | 0.209 |
| *g__Turicibacter* | BMI | -0.212 | <0.001 | 0.031 |
| *g__Megamonas* | BMI | 0.350 | 0.008 | 0.137 |
| *g__Allisonella* | BMI | 0.109 | 0.002 | 0.080 |
| **Phylum: *Fusobacteria*** | | | | |
| *g__Fusobacterium* | Alcohol consumption | 0.285 | 0.002 | 0.080 |
| **Phylum: *Proteobacteria*** | | | | |
| *g__Bilophila* | Cigarette consumption | 0.175 | 0.025 | 0.238 |
| *g__Haemophilus* | Cigarette consumption | -0.215 | 0.006 | 0.135 |
| *g__Haemophilus* | BMI | -0.178 | 0.006 | 0.135 |

# Genus with q value lower than 0.25 was presented.

* Multivariate analysis was performed using MaAsLin2 after adjustment for age, gender, recruitment region of participants, body mass index, family history of colorectal in first-degree relatives, history of colorectal polyp, use of nonsteroidal anti-inflammatory drugs, disease status (whether high-risk adenoma).

BMI: body mass index; FDR: false discovery rate.

## TABLE S2 Characteristics of the study population in external cohort.

| **Variable** | **Control (n=299)** | **CRC (n=299)** | **P value** |
| --- | --- | --- | --- |
| **Age, mean (SD)** | 60.9 (12.5) | 62.6 (9.8) | 0.386 |
| **Female/Male** | 115/134 | 97/152 | 0.123 |
| **BMI (kg/m^2^), mean (SD)** | 22.7 (3.0) | 23.0 (3.3) | 0.234 |
| **Cigarette consumption (Brinkman index), median (Q1 - Q3)** | 10.0 (0.0 - 400.0) | 240.0 (0.0 - 660.0) | **0.002** |
| **Alcohol consumption (grams per day), median (Q1 - Q3)** | 57.8 (0.0 - 316.0) | 38.6 (0.0 - 406.0) | 0.658 |

BMI: body mass index; CRC: colorectal cancer; Q1: the first quartile; Q3: the third quartile; SD: standard deviation.

## TABLE S3 Multivariate association between gut microbiota and lifestyle factors using MaAsLin2 Analysis in external cohort.

| **Genus^#^** | **Lifestyle factor** | **Coefficient** | **P value^*^** | **FDR** |
| --- | --- | --- | --- | --- |
| **Phylum: *Actinobacteria*** | | | | |
| *g__Bifidobacterium* | Alcohol consumption | -0.524 | 0.011 | 0.141 |
| *g__Bifidobacterium* | BMI | -0.454 | 0.022 | 0.220 |
| *g__Paratractidigestivibacter* | Alcohol consumption | 0.265 | 0.003 | 0.069 |
| **Phylum: *Firmicutes*** | | | | |
| *g__Granulicatella* | BMI | 0.416 | 0.024 | 0.238 |
| *f__Enterococcaceae;g__GGB33512* | BMI | -0.419 | 0.002 | 0.059 |
| *f__Clostridia unclassified;g__GGB33586* | BMI | -0.344 | 0.008 | 0.121 |
| *f__Clostridia unclassified;g__GGB3828* | BMI | 0.299 | 0.009 | 0.128 |
| *g__Hungatella* | BMI | -0.605 | 0.006 | 0.098 |
| *g__Intestinibacillus* | Alcohol consumption | 0.221 | 0.015 | 0.171 |
| *g__Intestinimonas* | BMI | -0.670 | 0.003 | 0.062 |
| *g__Eisenbergiella* | BMI | -0.606 | <0.001 | 0.018 |
| *f__Lachnospiraceae;g__GGB3463* | BMI | -0.397 | 0.004 | 0.075 |
| *g__Lacrimispora* | Cigarette consumption | -0.610 | 0.005 | 0.091 |
| *g__Faecalibacterium* | Alcohol consumption | -0.543 | 0.011 | 0.141 |
| *f__Oscillospiraceae;g__GGB9453* | BMI | -0.475 | 0.007 | 0.112 |
| *f__Oscillospiraceae;g__GGB9724* | BMI | -0.268 | 0.017 | 0.184 |
| *g__Phocea* | Cigarette consumption | -0.507 | 0.012 | 0.146 |
| *g__Solibaculum* | Cigarette consumption | 0.448 | 0.002 | 0.049 |
| *g__Erysipelotrichaceae unclassified* | Cigarette consumption | 0.496 | <0.001 | 0.005 |
| *g__Acidaminococcus* | BMI | 0.630 | 0.006 | 0.098 |
| *g__Megamonas* | Alcohol consumption | 0.528 | 0.002 | 0.060 |
| *g__Allisonella* | BMI | 0.582 | <0.001 | 0.005 |
| *g__Allisonella* | Alcohol consumption | 0.511 | 0.001 | 0.027 |
| *g__Dialister* | Alcohol consumption | -0.896 | 0.003 | 0.062 |
| *g__Megasphaera* | Alcohol consumption | 0.779 | 0.005 | 0.091 |
| **Phylum: *Fusobacteria*** | | | | |
| *g__Fusobacterium* | BMI | 0.751 | 0.004 | 0.077 |
| *g__Fusobacterium* | Alcohol consumption | 0.638 | 0.018 | 0.200 |
| **Phylum: *Proteobacteria*** | | | | |
| *g__Methylobacterium* | BMI | -0.525 | 0.006 | 0.098 |
| *g__Desulfovibrio* | BMI | -0.702 | 0.012 | 0.149 |
| *f__Aeromonadaceae;g__GGB9781* | BMI | -0.331 | 0.002 | 0.059 |
| **Phylum: *Spirochaetes*** | | | | |
| *g__Brachyspira* | Cigarette consumption | 0.379 | 0.005 | 0.091 |
| *g__Cloacibacillus* | BMI | -0.281 | 0.020 | 0.209 |

# Genus with q value lower than 0.25 was presented.

* Multivariate analysis was performed using MaAsLin2 after adjustment for age, gender, recruitment region of participants, body mass index, family history of colorectal in first-degree relatives, history of colorectal polyp, use of nonsteroidal anti-inflammatory drugs, disease status (whether high-risk adenoma).

BMI: body mass index; FDR: false discovery rate.

**Reference**

1. Yachida S, Mizutani S, Shiroma H, Shiba S, Nakajima T, Sakamoto T, Watanabe H, Masuda K, Nishimoto Y, Kubo M, Hosoda F, Rokutan H, Matsumoto M, Takamaru H, Yamada M, Matsuda T, Iwasaki M, Yamaji T, Yachida T, Soga T, Kurokawa K, Toyoda A, Ogura Y, Hayashi T, Hatakeyama M, Nakagama H, Saito Y, Fukuda S, Shibata T, Yamada T. 2019. Metagenomic and metabolomic analyses reveal distinct stage-specific phenotypes of the gut microbiota in colorectal cancer. Nat Med 25:968-976.

2. Blanco-Miguez A, Beghini F, Cumbo F, McIver LJ, Thompson KN, Zolfo M, Manghi P, Dubois L, Huang KD, Thomas AM, Nickols WA, Piccinno G, Piperni E, Puncochar M, Valles-Colomer M, Tett A, Giordano F, Davies R, Wolf J, Berry SE, Spector TD, Franzosa EA, Pasolli E, Asnicar F, Huttenhower C, Segata N. 2023. Extending and improving metagenomic taxonomic profiling with uncharacterized species using MetaPhlAn 4. Nat Biotechnol 41:1633-1644.
